# Appropriateness of drug prescriptions in patients with chronic kidney disease in primary care: a double-center retrospective study

**DOI:** 10.1186/s12875-022-01931-4

**Published:** 2022-12-14

**Authors:** Sonia Ruiz-Boy, Montserrat Rodriguez-Reyes, Joan Clos-Soldevila, Marina Rovira-Illamola

**Affiliations:** 1 Pharmacy Department, Hospital Clínic de Barcelona, St. Villarroel 170, 08036 Barcelona, Spain; 2 Primary Care Centre Comte Borrell, Consorci d‘Atenció Primària de Salut Barcelona Esquerra (CAPSBE), St. Comte Borrell 305, 08029 Barcelona, Spain; 3 Primary Care Centre Casanova, Consorci d‘Atenció Primària de Salut Barcelona Esquerra (CAPSBE), St. Roselló 161, 08036 Barcelona, Spain

**Keywords:** Primary care, Chronic disease, Geriatrics, Medical errors/patient safety, Frailty, Pharmacology/drug reactions

## Abstract

**Background:**

Chronic kidney disease (CKD) is a highly prevalent disease worldwide. A basic pillar for the management of a patient with CKD is the safe use of drugs. Inadequate dosing of medication or contraindicated drugs in renal impairment can lead to negative outcomes. The primary objective was to analyse the drug prescriptions of patients with CKD from two primary care centres to see if they were optimally adapted to the patient's estimated glomerular filtration rate (eGFR).

**Methods:**

A retrospective observational study was conducted in two urban primary care centres. The study period was between September–October 2019. Patients over 18 years of age, with established CKD and with an eGFR less than 60 mL/min/1.73m^2^ for at least three months were included. Their demographic data (age and sex) and clinical variables such as associated comorbidities, eGFR value were retrospectively registered. Finally, their medication plans were reviewed in order to detect: inappropriate prescribing (IP), defined as an incorrect dose/frequency or contraindicated drug according to the renal function of the patient; nephrotoxic drugs and drugs with a high sodium content.

**Results:**

A total of 273 patients were included. The most common patient profile was an elderly woman, polymedicated, with other concomitant diseases and with mild CKD. Two hundred and one IPs were detected, 13.9% of which were contraindicated drugs. Of all patients, 49.1% had been prescribed at least one IP on their medication plan, 93.8% had some potentially nephrotoxic drug and 8.4% had drugs with a high sodium content prescribed.

**Conclusions:**

Patients with CKD are at increased risk of medication-related problems. It is necessary to implement measures to improve the safety in the prescription of drugs in patients with CKD.

## Background

Chronic kidney disease (CKD) is defined, according to the latest update of the 2012 Kidney Disease Improving Global Outcomes (KDIGO) guidelines, as the presence, for at least 3 months, of an estimated glomerular filtration rate (eGFR) lower than 60 ml/min/1.73m^2^ or kidney injury markers [1]. CKD is classified into 5 stages according to the eGFR number, with stage 5 being the worst. The global prevalence of CKD is estimated as 9.1% and is up to 18% among those older than 65 years of age [2, 3].

The basic pillar for the management of a patient with CKD is the safe use of drugs. To do this, (i) the use of nephrotoxic drugs should be avoided, (ii) those drugs that can cause hyperkalaemia should be closely monitored, and (iii) the dose of drugs should be adjusted according to eGFR to guarantee their efficacy and safety [4]. Recommendations for drug adjustment include: reducing the dose, lengthening the frequency of administration, suspending treatment, or not initiating it [4].

One of the best-known nephrotoxic drug combinations in the primary care setting is a renin-angiotensin system inhibitor (RASI), a diuretic and a non-steroidal anti-inflammatory drug (NSAID), known as “Triple Whammy”. It has been observed that the use of the three pharmacological groups increases the risk of acute renal failure (ARF) by 31% compared to the dual therapy of an NSAID with a diuretic or a RASI [5, 6].

All this adds up to the importance of adopting a healthy lifestyle in the management of patients with CKD. For example, a low sodium diet is generally recommended, with a daily salt intake of less than 6 g (equivalent to 2.4 g of sodium). At this last point, those drugs that may contain significant amounts of sodium in their composition should be taken into account [7].

Thus, the control of CKD requires comprehensive and highly complex management, in which great attention must be paid to the patient’s treatment plan. Previous studies carried out both in the hospital setting and in primary health level have emphasized the need for a better adjustment in the treatment of these patients [2, 8–12].

Our objectives were: (I) to describe the profile of patients with CKD from two primary care centres; (II) to analyse drug prescriptions of those patients to see if they were optimally adapted to the patient’s eGFR; and (III) to assess the prescription of nephrotoxic drugs, including the combination "Triple Whammy", and drugs with high sodium content of those patients.

## Methods

A retrospective observational study was conducted in two urban primary care centres with an assigned population of 60,000 patients. The care centers were located in Barcelona, in Eixample Esquerra district.

The inclusion criteria were: patients over 18 years of age, with established CKD and with a GFR (estimated according to CKD-EPI) less than 60 mL/min/1.73m^2^ in an analysis carried out between September and October 2019 plus another analysis performed three or more months before. The exclusion criteria were: patients with no medication prescribed in the medication plan. Using the digital records of the centers, all patients who had undergone blood tests during those two months were searched for. Then, those patients that met the inclusion criteria, and not the exclusion criteria, were selected. The current prescriptions of the patients were analysed one month after the determination of GFR less than 60 mL/min/1.73m^2^.

In the electronic clinical record used in the health primary care centers analysed, medication plans and laboratory tests were coded. The doctor's summary of medical visits was free text. The electronic medication plan is the official document that includes both patient information (medication, dosage, duration), as well as the medical prescription for dispensing the medication at the pharmacy office. The dispensing of the medication is calculated based on the dosage entered into the computer system. When the patient presents kidney failure, the doctor should adjust the medication in the medication plan, if necessary.

The following variables were collected from the patients' medical records of the health primary care center: demographic data (age and sex), associated comorbidities, history of vascular or thrombotic events (defined as previous heart attack, unstable angina, ischemic stroke and pulmonary or venous thromboembolism), eGFR value, prescribed and current drugs and their dosage. To check if the medication was well adjusted for renal function and/or if there was any type of contraindication or precaution in CKD, the Summary of Product Characteristics of the drugs were consulted [13]. The *Micromedex* [14] and *Uptodate* [15] databases were used as alternative consultation sources.

Drug prescriptions at an incorrect dose/frequency and/or contraindicated according to the renal function of the patient were classified with the term inappropriate prescribing (IP). Those patients who were prescribed nephrotoxic drugs were identified, including the “Triple Whammy” combination. For that, drugs with a high potential for nephrotoxicity were consulted in different CKD management guidelines [2, 7, 16]. Regarding “Triple Whammy”, the concomitant prescription of a RASI with a diuretic and an NSAID was recorded, excluding paracetamol, metamizole and acetylsalicylic acid at doses less than 300 mg due to their low nephrotoxic potential.

Patients who received drugs with high sodium content were also identified. Those are the drugs with a sodium content greater than 50 mg per unit [7]. For this, the composition described in the Summary of Product Characteristics of those drugs whose presentation was effervescent, dispersible or granulated, was consulted [13].

In the present study, quantitative variables with normal distribution according to the Shapiro–Wilk test were expressed as mean and standard deviation. Variables with asymmetric distribution were indicated as median and interquartile ranges. Qualitative variables were expressed as absolute value and proportion. There was no formal sample size calculation. The statistical software used was Excel® (from Microsoft Office Professional Plus 2019).

This study has been approved by the center's research ethics committee and complies with the basic principles of the Declaration of Helsinki. It was not considered necessary to request informed consent from the patients because the study is based on retrospective data generated by clinical practice.

## Results

### Demographic and clinical characteristics of the patients

In the period studied, a total of 2,103 blood tests with eGFR’s determination were performed in patients from both primary care centers. Out of all of them, 284 (13.5%) patients with an eGFR < 60 ml/min/1.73m^2^ were identified. Of these, 11 patients (3.9%) were excluded from the study because they had not previously presented an eGFR below the mentioned limit.

Table 1 describes the demographic and clinical characteristics of the patients. There were more women (58,2%) in the sample population. An 87.2% of the patients (n = 238) were 75 years or older. The majority of patients had mild CKD, along with other concomitant diseases. The most frequent comorbidities were hypertension (89.1%) and dyslipidaemia (61.3%). 32.6% (n = 89) of the patients had suffered a vascular or thrombotic event. Furthermore, most of the patients were polymedicated, with 85.7% (n = 234) taking ≥ 5 drugs.

**Table 1:** Clinical and demographic characteristics of 273 primary care patients with CKD

**Female gender**, n (%)	159 (58.2)
**Age **in years, median (IQR)	85.00 (75.0–90.0)
**Estimated glomerular filtration rate** in ml/min/1.73m^2^ (eGFR), median (IQR)	48.0 (39.0–53.0)
**Number of other comorbidities and previous thrombotic or vascular events per patient**, median (IQR)	4.0 (3.0–5.0)
Comorbidity or previous thrombotic/vascular event, n (%)	
Hypertension	244 (89.4)
Dyslipidaemia	168 (61.5)
Depression/anxiety	110 (40.3)
Diabetes mellitus (type 1 or 2)	109 (39.9)
Osteoporosis	79 (28.9)
Obesity	76 (27.8)
Atrial fibrillation	65 (23.8)
Cardiac insufficiency/aortic aneurysm	61 (22.3)
Cancer	61 (22.3)
Previous heart attack/unstable angina/venous or pulmonary thromboembolism	52 (19.0)
Chronic obstructive lung disease/asthma	46 (16.8)
Previous ischemic stroke	37 (13.6)
Neurodegenerative disease/dementia	36 (13.2)
Chronic hepatitis B or C	12 (4.4)
**Patients according to severity of eGFR category**, n (%)	
G3A (45–59 ml/min/1.73m^2^)	162 (59.3)
G3B (30–44 ml/min/1.73m^2^)	78 (28.6)
G4 (15–29 ml/min/1.73m^2^)	28 (10.3)
G5 (< 15 ml/min/1.73m^2^)	5 (1.8)
Dialysis, n (%)	1 (0.4)
**Chronic kidney failure not reported as an active problem in the clinical record**, n (%)	59 (21.6)
**Number of prescribed drugs per patient**, mean ± SD	8.8 ± 3.9

IQR: interquartile range; SD: standard deviation

The eGFR’s average was similar between women (43.2 ± 11.6 ml/min/1.73m^2^) and men (46.1 ± 11.2 ml/min/1.73m^2^). The older the patients, worst the renal function, observing the lowest eGFRs in patients older than 90 years.

In 59 patients (21.6%), CKD was not registered as a current clinical problem in the patient's medical history. In 76.3% of these cases (n = 45), it was a mild CKD grade G3A (45–59 ml/min/ 1.73m^2^).

### Prescription’s adequacy

A total of 201 IPs were detected. Almost half of the patients included in the study (49.1%) had at least one IP on their prescription. The characteristics of these patients were quite similar to those described for all patients. Table 2 shows the types of IP found and the drugs involved. Most (77.1%) of the IPs found were: inappropriate dose and/or frequency (n = 158) and prescription of a contraindicated drug in CKD (n = 28).

**Table 2:** Inappropriate prescriptions by the patient’s eGFR: types of IP and drugs more involved in the medication plan of patients with CKD

**Total number of IP**, n	201
**Number of IP per patient**, mean ± SD	0.7 ± 1.0
**Number of IP per patient**, n (%)	
0	139 (50.9)
1	89 (32.6)
2	30 (11.0)
3	11 (4.0)
4	1 (0.4)
5	3 (1.1)
**Patients with any IP**, n, (%)	134 (49.1)
**Type of IP**, n (%)	201 (100.0)
Inappropriate dose and/or frequency	158 (78.6)
Contraindicated drug	28 (13.9)
Inappropriate presentation (it contains a higher dose/intake than recommended according to the patient’s FGe)	15 (7.5)
**Drugs more involved in IP**, n (%)	
**According to ATC code**	
Lipid modifying agents, plain (C10A)	45 (22.4)
Other analgesics and antipyretics (N02B)	44 (21.9)
Angiotensin converting enzyme inhibitors, plain (C09A)	33 (16.4)
Blood glucose lowering drugs, excluding insulins (A10B)	19 (9.4)
Benzodiazepine derivatives (N05CD)	14 (7.0)
Preparations inhibiting uric acid production (M04AA)	11 (5.5)
Mineral supplements: calcium (A12A) plus Vitamin D and analogues (A11CC)	5 (2.5)
Antidepressants (N06A)	5 (2.5)
Others	25 (12.4)
**According to active substance with respect to the total IP**	
Paracetamol	43 (21.4)
Atorvastatin	28 (13.9)
Enalapril	28 (13.9)
Fenofibrate	15 (7.5)
Lormetazepam	14 (7.0)
Metformin	10 (5.0)
Allopurinol	9 (4.5)
Sitagliptin	8 (4.0)
Others drugs	46 (22.8)
**According to active substance with respect to the total number of patients’ study with the prescribed drug**	
Rosuvastatin	2/2 (100.0)
Fenofibrate	15/16 (93.8)
Lormetazepam	14/16 (87.5)
Alfuzosin	4/5 (80.0)
Cetirizine	3/4 (75.0)
Atorvastatin	28/48 (58.3)
Sitagliptin	8/19 (42.1)
Enalapril	28/79 (35.4)
Paracetamol	43/125 (34.4)
Allopurinol	9/49 (18.4)
Citalopram	5/29 (17.2)
Metformin	10/59 (16.9)

Table 3 details the relationship between each type of IP and the drugs involved. In general, the therapeutic groups most implicated in the discrepancies were: lipid modifiers, non-NSAID analgesics and angiotensin-converting enzyme inhibitors (ACEi). The most commonly prescribed drugs at a dose higher than the recommended dose were enalapril, atorvastatin, lormetazepam, and paracetamol. The drugs that were prescribed while contraindicated were mainly citalopram, alfuzosin and cholecalciferol.

**Table 3:** Most involved drugs according to type of IP in the medication plan of patients with CKD

**Total daily dose too high**, n (%)	127 (100.0)
Enalapril	28 (22.0)
Atorvastatin	28 (22.0)
Lormetazepam	14 (11.0)
Paracetamol	11 (8.8)
Metformin	9 (7.1)
Sitagliptin	8 (6.3)
Other drugs	29 (22.8)
**Contraindicated drug**, n (%)	28 (100.0)
Citalopram	5 (17.9)
Alfuzosin	4 (14.3)
Cholecalciferol	4 (14.3)
Other drugs	15 (53.5)
**Inappropriate dose and frequency**, n (%)	19 (100.0)
Paracetamol	17 (89.4)
Other drugs	2 (10.6)
**Inappropriate presentation**, n (%)	15 (100.0)
Paracetamol	15 (100.0)
**Initial dose higher than the recommended dose**, n (%)	7 (100.0)
Allopurinol	5 (71.4)
Other drugs	2 (28.6)
**Frequency too high**, n (%)	5 (100.0)

Table 4 also lists nephrotoxic drugs, including the "Triple Whammy" combination, as well as drugs with a high sodium content found in patients. Almost all of the patients (93.8%) had been prescribed some nephrotoxic drug. On the other hand, the “Triple Whammy” combination was found in a very low percentage of patients (0.7%). In 8.4% of the patients, drugs with high sodium content had been prescribed.

**Table 4:** Nephrotoxic drugs, high sodium drugs and drugs to be handled with caution in renal impairment in the medication plan of patients with CKD

**Patients with ≥ 1 prescribed nephrotoxic drug**, n (%)	256 (93.8)
**Number of prescribed nephrotoxic drugs per patient**, median (IQR)	2 (1–3)
**Total of prescribed nephrotoxic drugs**, n (%)	599 (100.0)
Angiotensin converting enzyme inhibitors/angiotensin II receptor blocker	193 (32.2)
Diuretic drug	180 (30.1)
Proton-pump inhibitor	163 (27.2)
Allopurinol	48 (8.0)
Nonsteroidal anti-inflammatory drug	6 (1.0)
Antibiotic/antiviral drug	5 (0.8)
Other drugs	4 (0.7)
**Patients with Triple Whammy combination**, n (%)	2 (0.7)
**Patients with ≥ 1 prescribed drug that contain high levels of sodium**, n (%)	23 (8.4)
Paracetamol (effervescent tablet)	11 (47.8)
Calcium + vitamin D supplements (effervescent tablet)	10 (43.4)
Other drugs	2 (8.8)
**Drugs to be handled with caution**, n (%)	27 (100.0)
Because they can cause adverse effects in renal impairment	22 (81.5)
Because there is no information of their use in renal impairment	5 (18.5)
**Most involved drugs to be handled with caution**, n (%)	27 (100.0)
Calcifediol	8 (29.6)
Calcium carbonate	5 (18.5)
Other drugs	14 (51.9)

In addition, 27 drug prescriptions whose Summary of Product Characteristics recommend caution in patients with CKD were detected (Table 4).

## Discussion

In our study, 49.1% of patients with IP have been detected. In similar studies, this percentage varies between 20.0–74.5% [10, 12, 17–20]. According to literature, the causes of IP may be associated with: (i) short consultation time per patient [11, 21], (ii) lack of knowledge, inadequate use of clinical practice guidelines [11], (iii) by doctor’s decision after assessing the risk–benefit relation [22] or (iv) due to discrepancies in the drug’s dosage according to the consulted guide or document [23].

In absolute numbers, most of the IPs detected corresponded to paracetamol, atorvastatin, enalapril, fenofibrate and lormetazepam. However, this fact is influenced by the high frequency of prescription of these drugs in a primary care setting. Regarding drugs prescribed in the patient’s study, the drugs with a higher percentage of IPs were fenofibrate, lormetazepam, atorvastatin, sitagliptin and enalapril. We have observed that drugs most frequently involved in IP vary according to the study, either at the primary care [10, 20], in inpatients [8, 9], outpatients clinics [24] or geriatric residences [25]. The drugs or family drugs most related to IP which coincide most among the studies carried out in the community setting were allopurinol, antihypertensives (mainly ACEi and angiotensin II receptor blockers), hypoglycemic drugs and lipid-lowering drugs [10, 12, 18–20].

It is concerning that most patients (93.8%) had prescribed nephrotoxic drugs. This is a known problem, worsening in patients older than 60–75 years [26, 27]. On the other hand the prescription of NSAIDs in these patients has been minimal. We believe that this could have been due to the fact that the association of these drugs with an increased risk of acute kidney injury [16] is well known by prescribers.

In our cohort, we observed that the most of patients as commented in the results section, were older women (75–90 years), with associated comorbidities, polymedicated and generally with mild CKD. These characteristics coincide with those found in other studies also carried out in primary care setting [10, 11]. The profile differs from outpatients treated by nephrologists, the study by Laville et al. [24] showed that they were mostly men, younger (61–76 years) and with moderate-severe renal failure. This, together with multimorbidity and polypharmacy, makes them very prone to suffering from adverse effects due to the accumulation of the drug [17, 28].

In our study, only 2 cases of the “Triple Whammy” combination were found. In a study carried out in Catalonia, it was observed that the frequency of prescribing “Triple Whammy” would be 1.04 cases per 100 inhabitants [6], this frequency coincided with our study. In our area, an annual frequency of 3.4 cases of admissions due to acute kidney injury per “Triple Whammy” has been observed for every 1,000 drugs involved in “Triple Whammy” users. There is less consensus on whether the prescription of two of the three drugs that make up the combination would already cause acute kidney injury with the same frequency [5, 6].

An 8.4% of the patients had been prescribed a drug with a high sodium content. Although this is a small number of cases, these could have been avoided. That is because most of active ingredients have pharmaceutical presentations with lower sodium content. High sodium intake in patients with kidney disease has been associated with progression of kidney disease and increased use of antihypertensive drugs [29, 30].

For the adjustment of many drugs commonly prescribed in primary care, physicians tend to pay more attention to clinical markers such as heart rate, blood pressure or blood glucose than to kidney function [10]. Even so, the decrease in eGFR can disturb the metabolism and excretion of the drug [28], whose clinical consequences are difficult to predict [21]. However, there have been reports of hospitalizations of people with CKD due to adverse effects because of drug or their metabolites accumulation [23, 30, 31], as well as increased mortality [31]. Those negative outcomes could have been avoided by monitoring renal function and optimally adjusting the drug posology [22].

Finding solutions to improve drug adjustment in CKD patients is essential. Interventions, both computerized and manual, and education for professionals have been shown to significantly reduce IP. However, the greatest reduction is found in interventions by pharmacists or other clinical experts [22]. It has been shown with a high level of evidence that pharmacists can help improve control in other chronic diseases, such as blood pressure in patients with hypertension or glycosylated haemoglobin (HbA1c) in diabetic patients [32].

In a non-negligible percentage of patients (21.6%), the diagnosis of CKD was not registered as an active health problem in the patient's medical history. This issue has been reported in other articles [10, 11, 33]. This could contribute to suboptimal management in the care of these patients [11]. Pharmaceutical intervention can also help identify those CKD patients not reported in the system, which could help improve their medication plans [11, 33, 34]. In a study, it has been shown that the intervention of clinical pharmacist recommending the adjustment of drugs to the primary care physician is effective, and can contribute to a reduction in adverse effects and the costs derived from them [11].

In our primary care centers, there is a prescription assistance system integrated into the patient's medical history. This system shows alerts about antidiabetic drugs contraindicated in renal failure (alerts are linked to the analytical parameters of the patient's renal function) and prescription of the “Triple Whammy” combination. This system has surely contributed to the reduced number of IPs of these drugs detected in this study. It should also be noted that the clinical pharmacist performs a clinical validation of all direct-acting anticoagulant prescriptions, checking if their dosage is adjusted to the weight, age and eGFR of the patient. It has probably contributed to the fact that no IP was detected in this group of drugs. To improve the safety in drug prescription in patients with CKD, it would be desirable to extend the computerized alert system to the drugs with more IP detected in this study, as well as the involvement of pharmacists in the review of drugs with higher risk.

It is known that according to the equation used to calculate renal function, the prevalence of IP can vary. Although most dose adjustment guidelines were developed according to the Cockcroft-Gault (CG) equation, the CKD-EPI equation is more used by nephrologists to classify and monitor renal function in patients with CKD [10, 24]. It has been observed that the highest IP indices are obtained when the CKD-EPI equation is used, in comparison with the CG or the CKD-EPI adjusted for body surface area [24]. However, it is not clear if this difference would be significant [10]. On the other hand, in patients with extreme obesity, it is important to adjust the CKD-EPI equation by body surface area [24]. However, we were unable to adjust it because we did not have up-to-date weight and height values ​​in most cases.

Due to the retrospective nature of the study, we didn’t conduct interviews with the patients. Consequently, it was not possible to detect those drugs or herbal preparations self-administered by the patient with high nephrotoxic potential [35], as well as data on adherence. Also, all retrospective studies are susceptible to bias because they depend on data entered into a clinical database and not collected for research fulfilling the specific requirements of the study. Another limitation of the study is that the clinical consequences associated with the detected IPs were not collected.

As for strengths, we provide evidence from real clinical practice on a topic not very developed in the primary care setting.

We think that our results can be generalized to other primary care centers in the same health care area, since the profile of attended patients is the same, use the same electronic prescribing system, and similar consultation time per patient.

For the same reasons as those just stated, we do not believe that the results we would find now post-COVID pandemic would significantly differ from those we found then. The rate of IP could even have increased slightly due to the saturation of workload that there has arisen during some periods in primary care centers.

In the future, a prospective study could be carried out to see to what extent the pharmacist's recommendations on adjusting the medication plan are accepted by the prescribing physician and/or patient. Finally, since the study was conducted before COVID-19 pandemic, it would be interesting to recreate it now to see if as a result of COVID-19 the rate of IP has changed.

## Conclusions

In about half of the patients in our study, some medication was prescribed at a dose and/or frequency that were not adequate for their renal function. The consequent accumulation of the drug/metabolite can lead to negative outcomes (hospitalization or death). It is necessary to implement measures to improve the safety in the prescription of drugs in patients with CKD. The health centre pharmacist can help optimize prescribing.

## Data Availability

The datasets generated and/or analysed during the current study are not publicly available due to confidentiality agreements but are available from the corresponding author on reasonable request.

## Abbreviations

ARF: Acute renal failure
ACEi: Angiotensin-converting enzyme inhibitors
CKD: Chronic kidney disease
CG: Cockcroft-Gault
eGFR: Estimated glomerular filtration rate
HbA1c: Glycosylated haemoglobin
IP: Inappropriate prescribing
KDIGO: Kidney Disease Improving Global Outcomes
NSAID: Non-steroidal anti-inflammatory drug
RASI: Renin-angiotensin system inhibitor
